# A Framework for Examining Social Stress and Susceptibility to Air Pollution in Respiratory Health

**DOI:** 10.1289/ehp.0900612

**Published:** 2009-05-12

**Authors:** Jane E. Clougherty, Laura D. Kubzansky

**Affiliations:** 1 Department of Environmental Health and; 2 Department of Society, Human Development, and Health, Harvard School of Public Health, Boston, Massachusetts, USA

**Keywords:** air pollution, social stress, spatial analysis, synergistic effects, urban community health

## Abstract

**Objective:**

There is growing interest in disentangling the health effects of spatially clustered social and physical environmental exposures and in exploring potential synergies among them, with particular attention directed to the combined effects of psychosocial stress and air pollution. Both exposures may be elevated in lower-income urban communities, and it has been hypothesized that stress, which can influence immune function and susceptibility, may potentiate the effects of air pollution in respiratory disease onset and exacerbation. In this paper, we attempt to synthesize the relevant research from social and environmental epidemiology, toxicology, immunology, and exposure assessment to provide a useful framework for environmental health researchers aiming to investigate the health effects of environmental pollution in combination with social or psychological factors.

**Data synthesis:**

We review the existing epidemiologic and toxicologic evidence on synergistic effects of stress and pollution, and then describe the physiologic effects of stress and key issues related to measuring and evaluating stress as it relates to physical environmental exposures and susceptibility. Finally, we identify some of the major methodologic challenges ahead as we work toward disentangling the health effects of clustered social and physical exposures and accurately describing the interplay among these exposures.

**Conclusions:**

There is still tremendous work to be done toward understanding the combined and potentially synergistic health effects of stress and pollution. As this research proceeds, we recommend careful attention to the relative temporalities of stress and pollution exposures, to nonlinearities in their independent and combined effects, to physiologic pathways not elucidated by epidemiologic methods, and to the relative spatial distributions of social and physical exposures at multiple geographic scales.

There is growing interest within environmental health in disentangling effects of clustered social and environmental exposures and exploring potential synergies among these exposures (Gee and Payne-Sturges 2004; Morello-Frosch and Shenassa 2006; O’Neill et al. 2003, 2007a, 2007b; Weiss and Bellinger 2006). This interest stems from the observation that social stressors (i.e., violence, poverty) and environmental exposures (i.e., traffic-related and industrial air pollution) are often spatially correlated and may cluster in lower-income communities, often in locations with lower property values along highways and industrial corridors (Bullard 1990; Graves 1988). Traffic-related air pollution is a complex chemical mix (Schauer et al. 2006), often spatially associated with noise, poverty, and other stressors (Wheeler and Ben-Shlomo 2005). Social stressors may lead to poor health outcomes directly or may increase susceptibility to physical exposures, such as air pollution, through alterations in immune function and biological systems (McEwen and Seeman 1999). Thus, the most pollution-exposed communities may also be more susceptible because of higher prevalence of social stressors (Lipfert 2004). This potential for spatial confounding and effect modification by social stressors presents a challenge to air pollution epidemiology. Disentangling social and physical environmental risks (U.S. Environmental Protection Agency 2008), understanding synergies among these exposures, and identifying modifiable exposures are particularly important for understanding how best to protect susceptible populations and improve overall population health.

There is significant evidence linking traffic-related pollution exposure to respiratory illness. Chronic exposure to traffic-related and particulate air pollution among adults has been linked to upper respiratory tract inflammation (Hiltermann et al. 1997), chronic obstructive pulmonary disease (COPD) (Schikowski et al. 2005), lung cancer (Vineis et al. 2007), and premature mortality (Dockery 1993; Pope et al. 1995). In children, traffic-related pollution exposures, often indicated by residential proximity to major roads, has been associated with airway hyperresponsiveness (Jang et al. 2003), wheeze (Ryan et al. 2007), asthma (McConnell et al. 2006), reduced lung function (Pekkanen et al. 1997), and asthma-related hospitalizations (Lin et al. 2002; Moore et al. 2008). There is now increased interest in understanding cardiovascular effects (Peters 2005; Rückerl et al. 2006), identifying causal constituents (Lanki et al. 2006), and characterizing susceptible sub-populations (O’Neill et al. 2007b).

Psychological stress results when external demands exceed an individual’s perceived abilities and resources to meet those demands (Cohen et al. 1995) and has been linked to respiratory disease and other illness. Limited but growing epidemiologic evidence indicates that psychological stress may also alter susceptibility to physical exposures, such as air pollution. This work examined social–environmental interactions in respiratory and cardiovascular disease, such as stress-related modification of traffic-related air pollution effects on asthma etiology (Clougherty et al. 2007) or exacerbation (Chen et al. 2008). There is also evidence of stress-related modification in the relationship between bone lead level and hypertension among older men (Peters et al. 2007). In the same data set, we recently identified modification by stress in the relation between bone lead and heart rate variability (Clougherty JE, Peters JL, Schwartz J, Kubzansky L, unpublished data). One possible pathway for these differential susceptibilities is allostatic load (McEwen and Seeman 1999). Allostasis (literally, “achieving stability through change”) refers to the body’s ability to adapt to transient stressors and exposures. Over time, chronic psychological stress and maladaptive behaviors (e.g., poor diet, sleep deprivation) (McEwen 2006) may impair the body’s ability to maintain allostasis, producing wear and tear on bodily systems, compromised immune function, and enhanced general susceptibility (McEwen 2006; McEwen and Seeman 1999), and enhancing responsivity to environmental toxicants including air pollution. For example, some toxicologic evidence suggests permanent alteration in hypothalamic–pituitary–adrenal (HPA) function associated with chronic stress and lead exposures (Virgolini et al. 2005, 2006). However, some biological systems involved in maintaining allostasis may also be worn down by repeated exposure to physical toxicants, as particle exposures may influence HPA function (Sirivelu et al. 2006). Finally, some air pollutants and psychosocial stress may independently affect common physiologic processes such as oxidative stress (Fugisawa 2005) or inflammatory cell [immunoglobulin E (IgE)] production (Diaz-Sanchez et al. 2000; Nel 2001).

More studies have explored air pollution effect modification by socioeconomic status (SES) than by stress. Given other evidence of greater stress-related illness and susceptibility among persons with lower SES (Kubzansky et al. 2000; Miller and Chen 2007; Seeman et al. 2004), heightened air pollution responses with lower SES may be mediated through stress-related pathways. The American Cancer Society (ACS) studies reported stronger effects of air pollution on respiratory, cardiovascular, and all-cause mortality among lower-SES persons (Krewski et al. 2000), supported by other studies reporting stronger air pollution–health associations among less-educated adults (Finkelstein et al. 2003; Hoek et al. 2002; Pope 2002). Time-series studies have indicated that lower-SES persons display stronger associations between short-term air pollution exposures and health (Jerrett et al. 2004; Lin et al. 2004; Martins et al. 2004), although not consistently (Zanobetti 2000). These studies, however, have not explored specific components of SES that may be responsible for susceptibility effects, although stress has been proposed as a key component.

Several theoretical perspectives suggest that social, economic, and psychological disadvantage may cluster (Adler and Matthews 1994; Evans and English 2002; Link and Phelan 2000; Shonkoff and Phillips 2000). Fundamental causes theory, for example, describes social conditions (e.g., socioeconomic position) as determinants of resource access and psychological and behavioral experience, shaping disease risk (Link and Phelan 1995). Although applicable to combined social and physical exposures, these theories have not addressed methodologic issues (e.g., spatial correlation, confounding, measurement bias) or interpretation challenges arising in combining theory, methods, and data across disciplines. Like air pollution, low SES and stressors (e.g., noise) may be elevated in urban settings along highways and industrial areas, and methods are needed to disentangle effects of clustered social and physical stressors and to investigate potential synergies. One model (Morello-Frosch and Shenassa 2006) captures our key constructs of interest: *a*) varied stressors at the individual and community level contribute to individual chronic stress; *b*) chronic individual stress is embodied as allostatic load, or physiologic wear and tear, influencing individual susceptibility; and *c*) stress-borne physiologic susceptibility may shape response to environmental exposures, including internal dose, resiliency, likelihood of developing a given illness, and ability to recover from it.

Like other models combining social and physical exposures (deFur et al. 2007; Gee and Payne-Sturges 2004; O’Neill et al. 2003), the Morello-Frosch model acknowledges that some community-level stressors are also physical hazards (e.g., poor housing quality), complicating distinctions between effects of psychosocial and physical factors. This model frames our discussion, although it is outside the scope of our review to describe the myriad possible social, political, and economic forces that contribute to individual stress. Instead, we focus our attention on individual-level stress and pollution susceptibility, given that higher-level stressors need be individually embodied to modify pollution effects through stress-specific pathways.

We build on conceptual models of social–environmental interaction by describing key methodologic and conceptual issues that arise when merging data and methods from across environmental and social science traditions. We hope to foster more integrative approaches toward understanding combined health effects of social and physical environmental exposures. We attempt to synthesize the literature to date on the following:

- Effects of stress on respiratory health and susceptibility
- Epidemiologic and toxicologic evidence of synergistic effects of stress and pollution
- Pathways through which stress may influence pollution susceptibility
- Methodologic challenges in merging data and methods from environmental and social sciences.

We aim to provide a template for researchers aiming to rigorously consider combined effects on health across disciplines. We hope to support the growing literature targeted at disentangling effects of spatially clustered social and environmental exposures and exploring potential synergies among these exposures.

## Physiologic Effects of Psychological Stress on Respiratory Health and Susceptibility

A long history of research links psychological stress and respiratory health. In *Treatise on Asthma*, Maimonides (1135–1204) described the illness—“Whereas treatment of asthma is important, treatment of a patient as a whole is more important. For asthma the physician must be a doctor in every sense”—and described the importance of clean air and its interplay with emotions and hormones (Muntner 1963). In the 1930s, Hans Selye recognized links between chronic stress in laboratory rats and nonspecific susceptibility to respiratory distress and death (Selye 1936). More recently, chronic stress has been linked to asthma symptoms in cross-sectional studies (Oh et al. 2004), and prospective studies link caregiver stress to infant wheeze (Wright et al. 2002). Among asthmatic adolescents, SES has been associated with stress-linked immune mediators [interleukin (IL)-5, interferon-γ], an association mediated by stress and control beliefs (Chen et al. 2003). Acute stressors (e.g., violent events) may trigger asthma episodes (Sandberg et al. 2000, 2004), and older individuals with greater psychological distress and anger display faster lung function decline over time (Kubzansky et al. 2002).

Distinctions between acute (short-term, generally lasting days to weeks) and chronic (longer-term, lasting weeks to years) stress is not always clear. Some severe acute stressors (e.g., sexual violence, or combat) can produce chronic stress effects (i.e., post-traumatic stress disorder). Assessing stress chronicity is important, however, as acute and chronic stress can produce substantively different physiologic sequelae, and the predominance of acute versus chronic stress experience in a given period may shape observed relationships between stress and susceptibility. Acute stress is linked to sympathetic innervation, including increased ventilation and bronchodilation, plausibly masking some short-term effects air pollution effects (e.g., acute irritation, broncho constriction). Conversely, chronic stress can produce wear and tear on bodily systems, weaken immune function, and increase pollution susceptibility. Because of these very different physiologic sequelae, careful consideration of stress chronicity and of the relative chronicities of stress and pollution exposures is critical in accurately characterizing their interplay and in interpreting interactions.

### Acute stress, sympathetic regulation, and the fight-or-flight response

Sympathetic nervous system activation under acute stress produces the immediate, short-term fight-or-flight response. Neural synapses produce catecholamines (e.g., epinephrine and nor-epinephrine or adrenaline and noradrenaline), and catabolic functioning breaks down metabolites for physical activity and energy expenditure. Glucocorticoids are produced, including cortisol, increasing heart rate, ventilation, myocardial contraction force, arterial vasodilation to working muscles, vasoconstriction to nonworking muscles, and dilating pupils and bronchi. Under acute stress, parasympathetic nervous system–regulated activities subside (e.g., salivary and intestinal secretions for digestion and nutrient absorption, growth and repair). Frequent sympathetic nervous system dominance under repeated acute stress may interfere with growth and repair, especially important for children’s development, and suggests one pathway through which childhood stress may shape lifelong health and susceptibility.

### Chronic stress, immune function, and inflammatory response

Chronic stress may be characterized by recurrent acute stress or an inability to moderate acute stress responses (McEwen and Seeman 1999). Building on Hans Selye’s model of nonspecific susceptibility and allostatic load models of cumulative wear and tear, attention is increasingly being directed toward identifying biological mediators linking chronic stress to immune and endocrine function (Miller et al. 2002; Segerstrom and Miller 2004).

Chronic stress and associated negative emotional states (e.g., depression, anxiety, anger) may mediate immune and endocrine processes (Kiecolt-Glaser et al. 2002), with associations so consistent that some researchers have proposed reconceptualizing depression as dysfunction in HPA-axis regulation and stress response (Sternberg et al. 1992). Endocrine responses to chronic stress include dysregulation in production of catecholamines (epinephrine, norepinephrine), adrenocorticotropin, cortisol, growth hormone, and prolactin. Cytokines, particularly IL-6, stimulate corticotrophin-releasing hormone and HPA-axis activity, increasing plasma adrenocorticotropin hormone and cortisol (Hellhammer et al. 1997; Kirschbaum et al. 1995; Ockenfels et al. 1995; Seeman et al. 1997). Frequent activation of the glucocorticoid receptor by cortisol can lead to blunted glucocorticoid response (Miller et al. 2002), increased nuclear factor-κB signaling (Miller et al. 2008), and dysregulated catecholamine production (Glaser and Kiecolt-Glaser 2005).

Immune-linked inflammatory response may infleunce asthma and related airway disease. Asthma-linked parameters responsive to stress include IgE (Wright et al. 2004) and cytokine production (Chen et al. 2003) and respiratory inflammation (Umetsu et al. 2002). Early life chronic stress may alter T-helper (Th)1–Th2 immune cell balance (Umetsu et al. 2002), which is linked with childhood asthma, allergy, and inflammatory responses (Miller and Chen 2007).

### Impacts on common physiologic systems

Growing evidence suggests complex dose-dependent interactions among multiple pollutants (Mauderly and Samet 2009), mediated through the same mechanistic pathway, or some pollutants may potentiate effects of others. Similarly, stress may potentiate pollution health effects through immune and inflammatory processes, or stress and pollution may affect common physiologic systems. Both early childhood environmental exposures and stress-related catecholamines affect Th1–Th2 balance (Umetsu et al. 2002). Psychological stress (Epel et al. 2004), polycyclic aromatic hydrocarbons, cigarette smoke (Adcock and Ito 2005), and ozone (Fugisawa 2005) affect oxidative stress, which is linked to asthma and COPD (Rahman and MacNee 2000). Both stress and diesel exhaust particles are associated with elevated cytokines (e.g., IL-2, IL-6, and local IgE in nasal mucosa) (Diaz-Sanchez et al. 2000; Nel 2001). Some evidence suggests that stress may alter permeability of bodily membranes to chemical exposures, such that stress may alter systemic transport and chemical uptake into organs including the brain (Sinton et al. 2000), facilitating combined and synergistic effects of stressors and pollution on many bodily systems.

## Key Issues to be Addressed as This Work Progresses

### Careful attention to stress measurement

For environmental researchers, social epidemiologic theory and methods for stress measurement may be unfamiliar, but accurately understanding and applying these principles is necessary to produce meaningful analyses of interactions among stressors and pollution exposures. Careful attention to stress processes and distinction among the major components is needed to accurately capture and interpret stress effects on susceptibility. Moreover, because perceived stress is highly variable over time, it is important that measured stress periods are temporally appropriate to the pollution exposures and outcomes under study. The stress process is often described in three phases: *a*) the stressor (i.e., any event, condition, or external stimuli posing a physical or psychological challenge), *b*) stress appraisal (i.e., how one experiences, perceives, or interprets the event), and *c*) stress response (e.g., psychological and physiologic sequelae).

These phases are interdependent. A stressor appraised as benign or beneficial, rather than threatening, generally produces no stress response. Highly thorough stress assessments would measure each stage, which is rarely feasible. Most studies focus on a single, chronic stressor (e.g., caregiver burden) or one unlikely to be positively appraised (e.g., exposure to violence, natural disaster). Newer measures emphasize later stages of the stress process. Because of inconsistent findings with stressor measures (e.g., life event scales assessing major life changes) (Dohrenwend et al. 1993), more researchers today use perceived stress measures, which capture response to multiple stressors (Cohen et al. 1983, 1995). Recent evidence indicates, however, that subjective stress may poorly predict immune change (Segerstrom and Miller 2004); thus, another approach emphasizes negative affect (e.g., anxiety, depression) as a cumulative indicator of mental distress and psychosocial stress. Negative affect may serve as a common final pathway for multiple psychological stressors, possibly providing a more stable indicator of cumulative stress and susceptibility (Kubzansky et al. 2000; Seeman et al. 2002).

Other approaches include using bio-markers—hormones or immune markers associated with physiological stress responses, including glucocorticoids or cytokines (Miller et al. 2002). Although many physiologic systems are influenced by acute and chronic stress, there is relatively little consensus on optimal biomarkers to capture physiologic changes with acute or chronic stress. Formerly, corticosteroids (e.g., cortisol) in blood or saliva were emphasized as markers of HPA-axis activity, although stress-related HPA function changes lead to cortisol dysregulation (via glucorticoid resistance and HPA regulation), not simply increased cortisol production. Thus, cortisol can be difficult to interpret and better indicates acute rather than chronic stress. Recent research emphasizes indicators of glucocorticoid resistance and neuroendocrine signaling (Miller et al. 2008). Other evidence suggests that C-reactive protein (Miller G, personal communication) and tumor necrosis factor-α (Clougherty JE, Rossi CA, Lawrence J, Long M, Diaz E, Koutrakis P, et al., unpublished data) may capture chronic stress in rats. Although no single biomarker is appropriate for all applications (Brunner 2007), suites of physiologic parameters have been developed to represent allostatic load in humans, including indicators of cardiovascular function, metabolism, cholesterol, glucose metabolism, HPA-axis function, and sympathetic nervous system activity (Kubzansky et al. 2000; Seeman and Singer 2002). Several studies document chronic stress effects on cardiovascular risk indicators (abdominal obesity, elevated serum triglycerides, low high-density lipoprotein cholesterol, glucose intolerance, elevated blood pressure) (Brunner et al. 1997), known collectively as metabolic syndrome, and may provide a method for capturing cumulative stress effects on cardiovascular and systemic function.

### Relative temporality in stress and pollution exposures

Generally, stressors must precede or be contemporaneous with pollution exposures to plausibly modify their effects. The actual periods of stress and pollution exposures, their overlap, and temporal relation to health outcomes, deserve careful attention, because temporal exposure misclassification may nullify or even reverse the directionality of observed interactions. For example, if stress exposure occurs after or very late in a pollution exposure interval, interpreting observed interactions is problematic. If perceived stress is stable over time, the report will accurately reflect stress during the hypothesized period of susceptibility. If perceived stress varies over the period, however, and respondents compare current stress to prior experiences, then individuals reporting high stress likely experienced relatively lower stress previously, during the pollution exposure period. Such temporal misclassification may significantly confound results, and researchers should ensure that stress and pollution measurement periods support a plausible physiologic interaction in relation to the health outcome of interest.

### Spatial correlations among social and physical exposures

Both social and physical exposures vary across communities, and lower-income and minority communities may be disproportionately exposed to both (Evans and English 2002; Lupien et al. 2001; Morello-Frosch and Shenassa 2006). Strong spatial covariance among stress, SES, and pollution has confounded geographic information systems (GIS)–based air pollution epidemiology, and spatial epidemiologists are challenged to differentiate health effects of traffic-related pollution from those of spatially correlated noise, stress, or poverty. Evidence suggests that roadway noise, a spatial stressor correlated with pollution, increases heart rate among adults and children (Belojevic et al. 2008; Belojevic and Saric-Tanaskovic 2002), and living in high-traffic areas predicts higher stress, lower self-reported health, and depressive symptoms (Gee and Takeuchi 2004), effects distinct from those of pollution per se.

Because of spatial autocorrelation and possible confounding, accurate fine-scale exposure assessment for both stress and pollution is critical. GIS-based exposure models, now common in environmental epidemiology, should be validated to the spatial extent of the cohort examined, and spatial patterns in stressors and pollution, at multiple levels, should be carefully considered to avoid spatial misclassification and confounding. Social–physical correlations may vary by region or context, and the appropriate geographic scale of analysis may vary as well. The 1995–1997 Health Survey for England found that in urban areas, lower-SES households experienced worse air quality, although the opposite was true in rural regions. Air quality and SES independently predicted lung function in both settings, with greater pollution susceptibility among lower-SES men, but not women (Wheeler and Ben-Shlomo 2005).

Although some neighborhoods may experience higher average levels of pollution and stressors (variation across neighborhoods), one should not assume that individuals or residences within these neighborhoods are relatively more exposed to both (variation within neighborhoods). Individuals living closer to highways likely have higher pollution exposures than do other community residents but are not necessarily more exposed to violence or family stress. Similarly, U.S. census tracts, selected to be relatively homogeneous for key sociodemographic indicators, may be a meaningful unit of analysis for some social and economic factors (Subramanian et al. 2005). All persons within a census tract, however, do not have similar traffic-related pollution exposures, which vary dramatically within 50–200 m of major roadways (Zhu et al. 2002).

### Nonlinear, threshold, and saturation effects

There is evidence linking stress and air pollution, separately, to asthma onset and exacerbation. Because both exposures independently influence respiratory health, we might expect that with especially high levels of either exposure, exacerbations likely occur, regardless of the second exposure. Effectively, very high exposures may overpower any potential interaction effects.

Potential saturation effects call for careful attention to the exposure range observed in any study, relative to normal exposure levels, to inform whether interactions should be expected and how to interpret observed interactions. For example, our group reported that asthmatic children of families reporting higher fear of violence showed less symptom improvement with allergen-reducing indoor environmental interventions (Clougherty et al. 2006). Counter to initial hypotheses, this result suggested saturation effects in our very high-exposure public housing cohort. Either fear of violence or allergen exposures may have been high enough to independently induce or maintain symptoms.

### The need for toxicologic research

Because of strong potential confounding between stressors and pollution, epidemiologic methods alone are unlikely to fully differentiate their effects or resolve spatial confounding. Additionally, chronic air pollution exposure may contribute to HPA-axis stimulation and stress processes (Sirivelu et al. 2006). Thus, experimental studies using well-developed animal models of social stress (e.g., monkeys, rats) are needed to delineate these effects. Toxicologic data can improve causal inference and interpretability by randomizing exposures, controlling their temporality and intensity, and, importantly, can help elucidate physiologic mechanisms for stress-pollution interactions.

Several authors have investigated stress as a modifier of neurologic effects of lead in animal models. Cory-Slechta et al. (2004) examined separate and combined effects of restraint stress and lead in pregnant dams on corticosterone, neurotransmitter levels, and behavioral learning. They found significant interactions, generally more prominent in female offspring, and noted that lead alone influenced corticosterone, suggesting that lead may influence later-life susceptibility (Virgolini et al. 2005, 2006). They observed strongly altered HPA-axis function with both lead and stress, separately and in combination, providing a model for better understanding the etiology of conditions linked to both exposures and potentially mediated through HPA function, including obesity, hypertension, diabetes, anxiety, schizophrenia, and depression (Rossi-George et al. 2009).

We recently compared short-term respiratory response to concentrated fine particulate air pollution (CAPs) among chronically stressed and unstressed rats. Using the social dominance paradigm, a rat model of social stress (Hotsenpiller and Williams 1996; Luciano and Lore 1975), we introduced male test rats individually into the cage of an older, larger male, over an 8-week period, to establish chronic stress conditions. Test animals were exposed to controlled CAPs for 5 hr on days after stress exposures. Across four exposure groups [stress/CAPs; stress/filtered air (FA); nonstress/CAPs; nonstress/FA], animals exposed to both stress and CAPs displayed elevated C-reactive protein and altered respiratory function relative to other groups, with higher frequencies and briefer pauses after inspiration and expiration, suggesting a shallower, more rapid breathing pattern (Clougherty et al. 2008).

### Experimental studies involving humans

Experimental studies have identified physiologic parameters associated with stressors in humans (exam stress, public speaking). Although these studies can examine only short-term, relatively benign stressors, they do allow for controlled, randomized stressor exposures that are not feasible in the community setting. Fiedler et al. (2005) examined combined effects of stressors (public speaking, math challenge) and oxidation by-products of indoor volatile organic carbons and ozone, prevalent in office settings, on cortisol, respiratory function, and symptoms (anxiety, eye/nose irritation, respiratory symptoms) at multiple time points. They found that stress predicted anxiety symptoms and cortisol, outweighing any potential interaction with pollution (Fiedler et al. 2005). Such experimental studies cannot mimic the lifelong stress and pollution exposures associated with low SES but help to elucidate some mechanisms for stress-related susceptibility and, importantly, allow for the separation of persistently correlated exposures, otherwise difficult to delineate.

### Pollution and pollution sources as psychosocial stressors

Health geographers influenced by the environmental justice movement have long understood that neighborhood pollution sources, including highways, power plants, and smokestacks, can send strong messages to residents about the value of their health and well-being to the larger society (Bullard 1990). Indeed, the study of air pollution effects on health has historically considered its psychological effects (Colligan 1981; Strahilevitz et al. 1979), including psychosocial impacts of perceived exposure (Rotton et al. 1979) on vigilance (Horvath and Dahms 1971; Putz et al. 1977), test performance (Hore and Gibson 1968), irritation (Jones 1978), aggression (Rotton et al. 1979), behavior (Evans and Jacobs 1981), neurologic effects (Hore and Gibson 1968), and vision impairment (Lagerwerff 1963). More recent evidence indicates that air quality below regulatory guidelines can be detected by residents, with negative health consequences (Forsberg et al. 1997), although perceived air quality can also be influenced by disease status (Piro et al. 2008). Indeed, it can be difficult to distinguish health effects produced by the physical aspects of air pollution from its psychosocial health effects in communities near toxic sites (Elliot et al. 1993; Eyles et al. 1993), to separate both pollution-derived effects from those of other spatially correlated stressors, or to establish directionality. To better elucidate this complex synergy among exposure pathways, environmental exposure assessment and risk management may ultimately consider both tangible physical and psychosocial effects of pollution to fully understand the mechanisms linking air pollution, stress, and susceptibility.

## Additional Questions to be Explored

Many questions about stress-related pollution susceptibility remain. We raise several here, describing each only briefly, and hope these issues will be explored in detail in coming years.

### To which pollutants and health outcomes is stress-related susceptibility relevant?

A few studies demonstrate significant stress-related modification in air pollution effects on respiratory health, particularly asthma onset (Clougherty et al. 2007) and exacerbation (Chen et al. 2008; Clougherty et al. 2006). Stress may also modify relationships between bone lead and cardiovascular outcomes (e.g., hypertension) (Peters et al. 2007) and, in the same data set, heart rate variability (Clougherty JE, Peters JL, Schwartz J, Kubzansky L, unpublished data). A growing toxicology literature demonstrates stress-related susceptibilities to neurocognitive effects of lead and polychlorinated biphenyls, including neurologic damage and hippocampal function (Cory-Slechta et al. 2004). These vastly different exposures and outcomes demonstrating stress–pollutant interactions and the many physiologic systems affected by stress (e.g., sympathetic regulation, immune function, glucocorticoid production) suggest potential stress-related modifications in a wide range of pollutant exposures on health outcomes.

### Are these interactions different in illness onset than in exacerbation?

For preexisting illness, many factors may modify stress effects on illness severity and chronicity, including current medical treatment and comorbidities. The progression of preexisting illness, if influenced by stress, may also change the relevant temporalities, as later stress could plausibly modify recovery from environmentally derived illness. Thus, stress–pollution interactions may be even more complicated for existing illness, and careful attention to temporality and confounding (e.g., by medical treatment) is even more important. Some evidence suggests that stress may modify pollution effects in exacerbating preexisting asthma (Chen et al. 2008; Clougherty et al. 2006), although both studies produced associations opposite to hypotheses (stronger pollution effects with lower stress). More generally, attention to distinguishing processes related to illness onset from illness progression or exacerbation will be critical in assessing whether interactions are robust or should be expected in healthy populations.

### Are there critical periods for stress and pollution exposures across the life course?

Different diseases are relevant at different life stages. Asthma is among the most prevalent chronic illnesses of childhood, whereas cardiovascular illness generally afflicts older persons. Because the life-course distribution of illness varies considerably, relationships among stress, susceptibility, and illness likely also vary with age. Although stress can be toxic at any age, there may be critical periods, such as during early immune development, when it is particularly influential in shaping future susceptibility and disease risk (Evans and English 2002).

Parental stress has been used to examine stress exposures in young children, and research supports inverse associations for both mother’s SES and depressive states on children’s stress hormone levels (Lupien et al. 2000). Prospective studies have linked care-giver stress to infant wheeze (Wright et al. 2002), and maternal stress during pregnancy may influence immune function and health in neonates (Weiss and Bellinger 2006). Maternal air pollution exposures associated with low birth weight (Gouveia et al. 2004) may increase later childhood respiratory disease risk. Stress exposures during development may produce broad biological and psychological vulnerabilities, affecting neuroendocrine, immune, metabolic, and growth processes (Viltart and Vanbesien-Maillot 2007). Although not entirely immutable, these effects may have permanent and compounding consequences, especially if not addressed early (Cicchetti and Walker 2001; McEwen 2008).

### What are the roles of gender/sex and race/ethnicity in shaping stress-related susceptibility to pollution?

It is beyond the scope of this review to describe the myriad societal-level sources of stress. However, ongoing research in this area will be informed by the growing literatures on racism as a prevalent stressor, and on sex and gender differences in psychological and physiologic responses to stress and susceptibility.

Racial and ethnic minority groups in the United States are often differentially exposed to psychosocial stressors and physical contaminants in their homes, neighborhoods, and workplaces (Bullard 1990). The environmental justice movement has drawn attention to disproportionate pollution exposures in minority and low-income communities (Krieger 1990), but only recently has attention been directed to combined pollution exposures and greater susceptibility in these communities (Clougherty et al. 2007; Evans and English 2002; Morello-Frosch and Shenassa 2006; National Environmental Justice Advisory Council 2004). Experiences of racism as a stressor have been associated with negative physical and emotional health effects (Borrell et al. 2006; Clark et al. 1999; Krieger and Sidney 1996; Kwate et al. 2003; Landrine and Klonoff 1996; Thompson 1996) modified by coping practices and social support (Kwate et al. 2003). Experiences of racial discrimination predicted poorer birth outcomes among women with Arabic names in California after 11 September 2001 (Lauderdale 2006). There is also evidence of poorer health, including mortality, for U.S. persons than for foreign-born persons of the same ethnicity, especially Hispanics (Singh and Hiatt 2006). Effects of race and racism are not fully mediated through social class (Kwate et al. 2003) and are not generally attributable to genetic variation (Krieger 2005). Race-related stressors may contribute to “weathering” or physiologic wear and tear (Geronimus 1992; Goodman et al. 2005; Merritt et al. 2004). Hypertension onset, for example, may be four to seven times higher among low-SES than among high-SES African-American men (James et al. 2006). African Americans also have higher rates of cardiovascular illness (Pappas et al. 1993) and low birth weight (David and Collins 1997) relative to Caucasians with comparable education.

Sex and gender differences in stress and susceptibility may be significant. The distinction between gender (socially influenced behaviors) and sex (biology, including hormonal composition, sex organs, and anthropometric measures) is comparable with that between exposure and susceptibility. Sex differences have been observed in air pollution–health associations. After early childhood, more studies report greater risks among women and girls (Luginaah et al. 2005; Oftedal et al. 2008; Rojas-Martinez et al. 2007; Rosenlund et al. 2008), possibly explained, in part, by biological sex differences in lung capacity, hormonal status, inflammation, or airway particle deposition (Kim and Hu 1998; Kohlhaufl et al. 1999). Gendered social and behavioral patterns also matter. Sex stratification in the workforce and work-related exposures are well documented (Arbuckle 2006; Messing and Stellman 2006). Among children, play activities may influence ventilation rate, creating exposure differences between boys and girls (McConnell et al. 2002). Common pollution exposures metrics may be differently accurate by gender; residential exposures may better capture exposures for stay-at-home parents (usually mothers) than for working adults (Dietrich et al. 2008; Jedrychowski and Krzyzanowski 1989). Physiologic stress responses differ by sex (Sundaram et al. 2004; Taylor et al. 2000), as does observed effect modification by SES (Wheeler and Ben-Shlomo 2005), such that gendered stress assessment may be needed to fully understand stress-linked effect modification for air pollution.

### To what extent are the effects of SES attributable to stress?

As described, there is growing evidence of SES effect modification in the air pollution–disease relationship. ACS studies reported stronger health effects of air pollution among less-educated adults (Krewski et al. 2000), and time-series studies report the same (Jerrett et al. 2004; Kan et al. 2008; Lin et al. 2004; Martins et al. 2004), although these air pollution studies have not identified causal components of SES shaping susceptibility. Like air pollution, SES is a complex exposure mix accumulated over the life course, potentially including poorer-quality education; nutrition; housing; health care; health behaviors; pollution exposures in the home, community, and workplace; perceived social position; and other stressors. Indeed, newer variants of the allostatic load model incorporate primary (e.g., perceived stress, psychological response) and secondary (e.g., smoking, sleep, dietary behaviors) manifestations of stress (McEwen 2006). Growing evidence links low SES to perceived stress and distress (Adler et al. 1994; Miller and Chen 2007) and to biological markers of stress-related susceptibility (Lupien et al. 2001). Some of these studies used allostatic load models to link SES to distress and markers of disease risk (e.g., metabolic dysregulation) (Kubzansky et al. 2000). Other studies link allostatic load parameters to morbidity and mortality, suggesting that allostatic load may mediate relationships between SES and mortality, after accounting for demographic characteristics and physician-diagnosed illness (Seeman et al. 2004).

Among children, low SES may be a marker of early exposure to adversity. Recent studies link low childhood SES to epigenetic changes in messenger ribonucleic acid (mRNA) for the glucocorticoid receptor and toll-like receptor 4, indicative of proinflammatory profiles associated with asthma and allergic illness (Miller and Chen 2007). Low-SES children 6–10 years of age have shown higher basal cortisol levels than higher-SES children (Lupien et al. 2001). Children of parents reporting higher stress display physiologic hyperresponsiveness, and low-income children of depressed mothers show sex-specific physiologic responses: elevated cortisol in girls, HPA-axis hypoactivity in boys (Fernald et al. 2008). Children of depressed mothers can display altered adrenocorticotropic hormone and corticotropin-releasing hormone production into young adulthood (Ronsaville et al. 2006). Evidence suggesting that stress is a key mediator for SES effects on health is not universally endorsed, and associations between SES and stress-related biological changes are not always positive (Dowd and Goldman 2006). Authors have argued that psychosocial factors are inextricably bound to material factors in developed countries; therefore, the accumulated evidence does not unequivocally demonstrate an independent pathway by which psychosocial stress may link SES to physical health (Macleod and Davey Smith 2003). Other aspects of SES (e.g., nutrition, health care) may also influence pollution susceptibility, and methods are needed to determine their effects. We believe that the evidence to date is broadly suggestive of a link between SES and physiologic susceptibility, a significant portion of which may be explained by life stress. Continued research in this area is valuable, especially as it improves our understanding of the pathways through which physical exposures, including pollution, may differently impinge on the health of vulnerable populations.

## Conclusions

There is tremendous potential, although much work still to be done, in understanding combined effects of social and physical environmental exposures. These topics are exceedingly complicated. Accurately characterizing both social and physical exposures is a significant challenge that must be performed carefully, especially in light of potential confounding across the exposures of interest, before analyzing and interpreting interactions.

The issues we have highlighted here— temporal relationships between stressors and pollution; nonlinearity and saturation effects; spatial colinearity across exposures; age-related susceptibility and critical periods; and distinctions between processes related to illness etiology and exacerbation—will be critical in exploring social–environmental interactions. These factors are significant enough to distort observed associations (e.g., saturation effects may reverse interactions at high exposures; treatment can mask exacerbations of pre-existing illness). With sustained attention to these issues, we can work toward a clearer, richer understanding of complex effects among social and physical environmental exposures on health.
